# Fabrication and near-field visualization of a wafer-scale dense plasmonic nanostructured array†

**DOI:** 10.1039/c7ra13322g

**Published:** 2018-02-08

**Authors:** Jungheum Yun, Haemi Lee, ChaeWon Mun, Junghoon Jahng, William A. Morrison, Derek B. Nowak, Jung-Hwan Song, Dong-Kwon Lim, Tae-Sung Bae, Hyung Min Kim, Nam Hoon Kim, Sang Hwan Nam, Jongwoo Kim, Min-Kyo Seo, Dong-Ho Kim, Sung-Gyu Park, Yung Doug Suh

**Affiliations:** Advanced Functional Thin Films Department, Korea Institute of Materials Science (KIMS) Changwon 51508 Republic of Korea sgpark@kims.re.kr; Research Center for Convergence NanoRaman Technology, Korea Research Institute of Chemical Technology (KRICT) Daejeon 34114 Republic of Korea ydsuh@krict.re.kr; Center for Nanocharacterization, Korea Research Institute of Standards and Science Daejeon 34113 Republic of Korea; Molecular Vista San Jose CA 95119 USA; Department of Physics and Institute for the NanoCentury, KAIST Daejeon 34141 Republic of Korea; KU-KIST Graduate School of Converging Science and Technology, Korea University Seoul 02841 Republic of Korea; Jeonju Center, Korea Basic Science Institute Jeonju 54907 Republic of Korea; Department of Bio & Nano Chemistry, Kookmin University Seoul 02707 Republic of Korea; Center for Convergent Research of Emerging Virus Infection, Korea Research Institute of Chemical Technology (KRICT) Daejeon 34114 Republic of Korea; School of Chemical Engineering, Sungkyunkwan University Suwon 16419 Republic of Korea

## Abstract

Developing a sensor that identifies and quantifies trace amounts of analyte molecules is crucially important for widespread applications, especially in the areas of chemical and biological detection. By non-invasively identifying the vibrational signatures of the target molecules, surface-enhanced Raman scattering (SERS) has been widely employed as a tool for molecular detection. Here, we report on the reproducible fabrication of wafer-scale dense SERS arrays and single-nanogap level near-field imaging of these dense arrays under ambient conditions. Plasmonic nanogaps densely populated the spaces among globular Ag nanoparticles with an areal density of 120 particles per μm^2^ upon application of a nanolithography-free simple process consisting of the Ar plasma treatment of a polyethylene terephthalate substrate and subsequent Ag sputter deposition. The compact nanogaps produced a high SERS enhancement factor of 3.3 × 10^7^ and homogeneous (coefficient of variation of 8.1%) SERS response. The local near fields at these nanogaps were visualized using photo-induced force microscopy that simultaneously enabled near-field excitation and near-field force detection under ambient conditions. A high spatial resolution of 3.1 nm was achieved. Taken together, the generation of a large-area SERS array with dense plasmonic nanogaps and the subsequent single-nanogap level characterization of the local near field have profound implications in the nanoplasmonic imaging and sensing applications.

## Abstract 2

This paper presents the fabrication and near-field visualization of a wafer-scale dense plasmonic nanostructured array.

## Graphical abstract

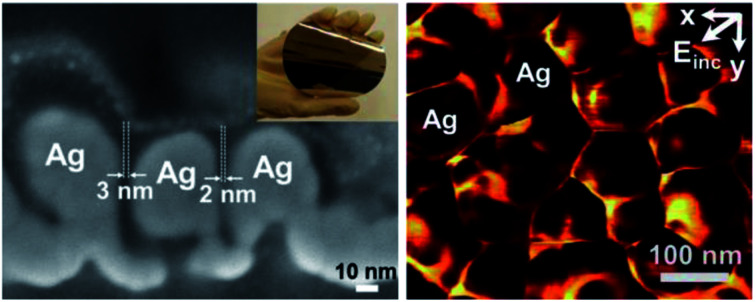

## Introduction

The development of surface-enhanced Raman scattering (SERS)-based techniques that identify and quantify trace amounts of analyte molecules is highly desired for practical applications in diverse areas such as environmental and food monitoring, photochemical reaction monitoring, and preventive point-of-care detection, and even in the early detection of warfare agents.^1–4^ Great efforts have been made to generate dense plasmonic nanogaps (also called hot spots) that give rise to enhanced SERS signals using nanofabrication techniques and chemical synthesis techniques.^5–13^ Nanoimprint lithography, for example, offers an excellent fabrication route to generating large plasmonic arrays with high throughput; however, this nanofabrication technique produces nanostructured arrays with a wide period of 200 nm (or 25 nanorods per μm^2^), which only moderately enhances the plasmonic field.^13,14^ Recently, a cost-effective maskless dry etching process representing a viable route to hot spot generation was applied to silicon to produce large substrates that supported metal-coated nanopillars. However, this nonlithographic technique also produced a low areal density of nanopillars, and capillary leaning effects must be applied to form plasmonic hot spots with nanoscale dimensions.^15^ We previously made high aspect ratio (HAR) Au nanopillar arrays with large gap distances between plasmonic nanopillars, and HAR Au nanopillars also need capillary leaning effect to form collapsed nanogaps.^16^ Therefore, a significant increase in the areal density of small nanogaps is required to enable sensitive and uniform SERS detection.

SERS arrays with high areal-density nanogaps require thorough characterization of the electric field distributions at the single nanogap level. The non-propagating evanescent nature of the near field presents several challenges to the high-resolution electromagnetic field mapping at highly confined plasmonic nanogaps. Far-field Rayleigh scattering has been used frequently to characterize the plasmonic response and correlate this response with the SERS signal intensity. However, no direct correlation between these signals has yet been identified,^17,18^ mainly because the spatial electric field distribution within the highly confined nanogaps is distinct from the spatial field distribution of the Rayleigh scattering that reflects the average field distribution surrounding the plasmonic nanostructures. Several studies have obtained near-field images of diverse plasmon modes created in isolated nanostructures and nanoassemblies.^19–24^ Near-field imaging of dense plasmonic arrays, however, requires further complicated system, such as optimized designs of harmonic demodulation or interferometric implementations, to remove the strong far-field background radiation arising from direct excitation of the sample in the diffraction-limited illumination area.^25–27^ Electron microscopy-based techniques, such as electron energy loss spectroscopy and cathodoluminescence spectroscopy, also provide near-field images. These techniques necessitate high-vacuum and high-irradiance power conditions.^28^

Here, we report on a simple fabrication method for generation of dense plasmonic nanogaps over a 4 inch surface area. Nanolithography-free SERS substrate with dense nanogaps exhibited a highly sensitive SERS enhancement factor (EF) of 3.3 × 10^7^ and uniform SERS response with the coefficient of variation (CV) of 8.1%. Nanoscale near-field characterization of individual nanogaps was carried out using photo-induced force microscopy (PiFM). The quantitative measurement of the near-field distributions at the single-nanogap level will provide practical guidelines for the rational design of metal nanostructures in the nanoplasmonic imaging and sensing applications.

## Results and discussion

**Fig. 1:**
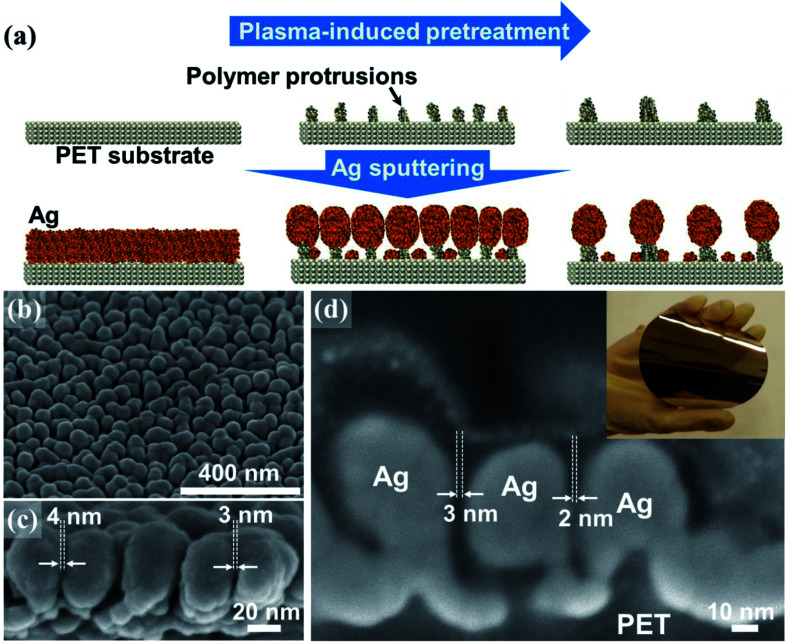
(a) Schematic diagram illustrating a nanolithography-free two-step fabrication process for preparing a dense plasmonic array, consisting of Ar plasma-induced treatment of the PET substrate and the subsequent deposition of Ag nanoparticle array onto the treated PET surfaces. SEM images (b and c) showing the detailed morphology of the dense Ag nanoparticle array deposited onto the polymer protrusions. (d) Focused ion beam-milled cross-sectional SEM image displaying the nanogaps formed between neighboring Ag particles. The inset in (d) shows a photograph of a 4 inch SERS substrate.

The fabrication method of low areal density of Au nanopillar array using maskless Ar plasma treatment to polyethylene terephthalate (PET) surface and Au evaporation were previously reported.^16^ The fabrication of the SERS arrays in this study also involved a two-step sequence comprising an Ar plasma-induced PET treatment and subsequent Ag sputtering onto the treated PET substrate (Fig. 1a). Key morphological features were achieved through the preferential self-assembly of the Ag nanoparticles on the polymer protrusions formed on the large PET substrate. Polymer protrusions frequently form on soft polymer surfaces exposed to plasma-induced ion irradiation.^36,37^ The formation of protrusions appears to result from complex surface dynamics, including plasma etching, redeposition, surface migration, and the agglomeration of volatile low molecular weight polymer chains generated by random ion-induced chain scission and recombination events.^38^ As the plasma treatment time was increased, the size and areal density of protrusions changed significantly due to coalescence among neighboring protrusions. The size evolution of the protrusions was accompanied by a reduction in their areal density and by an increase in the average distance between neighboring protrusions (ESI, Fig. S1†).

The periodicity of the polymer nanoprotrusions, which was a direct function of the plasma treatment time, was central to systematically controlling the areal density of Ag nanoparticles because the Ag particles grew preferentially on the protrusions (Fig. 1a). The formation of an individual Ag nanoparticle on a polymer protrusion was thought to proceed *via* rapid deposition of a relatively large flux of Ag species onto the topmost surface of the protrusion. Ag deposition onto the recessed PET surfaces between the protrusions, on the other hand, was significantly constrained by the local shortage of Ag species primarily due to the shadowing effects of adjacent Ag nanoparticles (ESI, Fig. S2†). Once the Ag sputtering process had terminated, an array of globular Ag nanoparticles was superimposed on the polymer protrusions. The globular geometry of Ag particles was ascribed to surface energy minimization effects. Scanning electron microscopy (SEM) images indicated that nanogaps formed at the junctions between neighboring Ag particles (Fig. 1b–d). The average number density of Ag particles was estimated to be approximately 120 particles per μm^2^ in the case of a 60 nm thick Ag nanoparticle array deposited onto a PET surface pretreated under plasma over 60 seconds. The gap sizes of ninety <15 nm gaps were precisely examined using focused-ion beam (FIB)-milled cross-sectional SEM (Fig. 1d and S3†). A high fraction (up to 45%) of nanogaps was observed to be ≤4 nm in size, with a mean of 5.6 ± 3.1 nm (ESI, Fig. S4†). It should be noted that the measured gap sizes might be overestimated due to the technical difficulties associated with ion milling of the samples across the centers of the Ag particles. Overall, the advantage of this fabrication technique lies in the fact that it is an easily accessible nanolithography-free approach to significantly increasing the density of plasmonic nanogaps across a 4 inch flexible surface area (inset in Fig. 1d). The UV-visible absorption spectra of the SERS arrays exhibited changes in the optical properties as a function of the Ag nanoparticle size (ESI, Fig. S5†). As the Ag nanoparticle size increased from 50 to 60 nm, the absorbance peak was red-shifted and broadened at longer wavelengths, indicative of strong plasmonic coupling between adjacent particles. Further increases in the Ag particle size beyond 60 nm resulted in a blue shift and resharpening of the absorbance peak, suggesting aggregation among adjacent particles. This observation agreed well with the SERS measurements, in which an array of 60 nm Ag particles displayed the most intense SERS signal (ESI, Fig. S6†). The SERS arrays studied here supported the use of 60 nm Ag nanoparticles on polymer protrusions that had been pretreated using a 60 second plasma treatment. We next examined the large-area signal uniformity of our SERS array using a 1.3 μm beam size. The SERS array was immersed in a 2 μM benzenethiol solution for one hour, followed by rinsing with ethanol several times. A microRaman microscope equipped with a 514.5 nm excitation source having a 1.3 μm beam size was employed to measure the SERS signals of the adsorbed benzenethiol molecules. The incident power and accumulation time were 150 μW and 10 seconds, respectively. The SERS spectra were measured at 9 positions across the entire 4 inch surface area (ESI, Fig. S7†). The obtained spectra exhibited a low degree of variation in the observed SERS intensities, with a standard deviation of 10.1%. The uniform SERS signal was attributed to both the large detection area (1.3 μm^2^) and the high areal density of plasmonic nanogaps formed among Ag particles (∼156 particles/1.3 μm^2^).

**Fig. 2:**
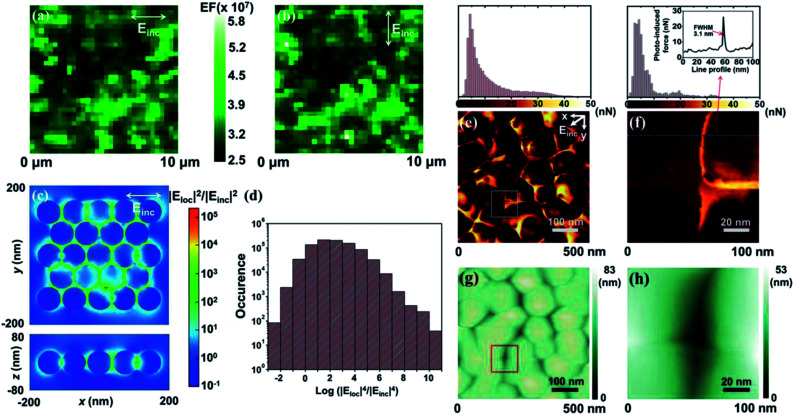
Spatially mapped SERS EF images (10 × 10 μm^2^ with 32 × 32 confocal spots) of a 2 μM benzenethiol-adsorbed SERS array under incident illumination, polarized either (a) parallel to the slow scan axis or (b) perpendicular to the slow scan axis. (c) Finite-difference time-domain simulation of the electric field distribution in the *XY*-plane (upper panel) and *ZX*-plane at *y* = 0 (lower panel). The *XY*-plane at *z* = 0 contained the centers of each Ag nanoparticle. (d) The calculated average electromagnetic enhancement. (e and f) PiFM images and (g and h) simultaneously obtained topographic images. The spatial resolution of PiFM was found to be 3.1 nm based on the determination of the FWHM, as shown in (f). The plasmonic array was illuminated with 532 nm incident light in (a)–(c), (e) and (f).

SERS intensity variations collected over small detection areas were predominantly dictated by the areal density of the plasmonic hot spots. A large number of hot spots produce small statistical fluctuations in the measured SERS intensities. We thus used a scanning confocal microscope with a small diffraction-limited diameter of 360 nm (corresponding to 0.1 μm^2^) to investigate the SERS signal uniformity. SERS mappings using a Raman mode at 998 cm^−1^ were carried out using the 2 μM benzenethiol-treated SERS arrays. The scanned area (10 × 10 μm^2^) was mapped over 32 pixels such that each pixel was 312 nm, approximately corresponding to the laser spot size. The resulting SERS EF was measured under an incident polarization either parallel to the slow scan axis (Fig. 2a) or perpendicular to that scan axis (Fig. 2b). A set of detailed measurements was collected under incident polarized light with an angular rotation of 45° (ESI, Fig. S8†). The very weak polarization-dependent SERS response was largely due to stochastic orientations of the high-density hot spots. Well-ordered non-spherical structures, by contrast, showed significant changes in the SERS enhancement and varied with the angle of the nanostructure's major axis, the nanoparticle polarizability (*α*), and the incident electric field (*E*_inc_) (EF ∝ [*αE*_inc_]).^39–41^ A numerical study revealed that a triangular trimer with a spherical geometry (*D*_3h_) exhibited a constant EF, whereas a linear trimer with a one-directional elongated geometry (*D*_∞h_) exhibited EFs that differed by several orders of magnitude, depending on the incident polarization angle.^42^ The weak anisotropy observed here is, thus, an important characteristic of ideal SERS arrays that generate a uniform SERS response irrespective of the incident polarization direction.

In support of the SERS measurements, we performed finite-difference time-domain (FDTD) calculations of the electric field across the SERS array (Fig. 2c). The randomly distributed Ag nanoparticles were modeled by generating random perturbations at the horizontal positions of 23 Ag nanoparticles in a triangular lattice. The standard deviation of the position perturbation was set to 2 nm. The diameter of the Ag nanoparticles and the periodicity of the triangular lattice were 68 and 72 nm, respectively, so that the particle size and average nanogap (4 nm) corresponded to FIB-milled cross-sectional SEM images. The array was illuminated, through the top *z*-axis, with 532 nm incident light that was linearly polarized along the *x*-axis. As expected, the field strength, |*E*_loc_|^2^/|*E*_inc_|^2^, was concentrated at the nanogaps, and the maximum field strength was 1.35 × 10^5^ at the smallest nanogap of 1 nm. The SERS electromagnetic enhancement could be roughly approximated as |*E*_loc_|^4^/|*E*_inc_|^4^, taking into account that the Raman shift was small compared to the spectral width of the plasmonic resonance. The average SERS electromagnetic enhancement, 〈|*E*_loc_|^4^/|*E*_inc_|^4^〉, was calculated by considering only the Ag shell surfaces in the horizontal plane, including the centers of the Ag nanoparticles (*z* = 0 plane). As a result, the average electromagnetic enhancement was estimated to be 2.4 × 10^7^, based on a shell thickness of 0.5 nm (Fig. 2d). These results agreed well with the experimentally obtained SERS EF.

Further, to elucidate the uniformity and magnitude of the plasmonic enhancement, it was necessary to probe the electromagnetic field distributions at individual nanogaps in the plasmonic nanostructures. Fig. 2e and g present the PiFM and topographic images (500 × 500 nm^2^), measured under ambient conditions. The topographic effect in the PiFM image was removed by quantitatively reconstructing the amplitude of the cantilever (Fig. S9 and S10†).^43–45^ The polarization vector of the incident electric field (*E*_inc_) contained both *x* and *y* components to efficiently generate induced dipole moments at randomly oriented nanogaps. The incident electric field also possessed a *z*-component parallel to the tip apex, which created an image dipole at the tip-apex. The obtained PiFM image exhibited a stronger near-field force at the nanogaps compared with that measured at the Ag surfaces. These results indicated conclusively that larger induced dipole moments were generated at the nanogaps. Fig. S11† illustrates the consistency of the results measured over different areas. Our experimental observations corroborated the established theoretical prediction that highly confined nanogaps contributed predominantly to the SERS enhancement.^46^ In agreement with our observation, a pair of smooth Au film-coupled nanostripes with a 1 nm gap have been reported to yield a fairly high plasmonic enhancement of 10^5^.^47^Fig. 2f and h exhibit a rescanned PiFM image and a topographic image (100 × 100 nm^2^), respectively, of the boxed areas shown in Fig. 2e and g. The spatial resolution of the PiFM image was determined to be 3.1 nm based on the full width at half maximum (FWHM) of the dotted line profile of the near-field force (Fig. 2f, upper panel). Such a high spatial resolution signified that the measured near-field force varied sensitively with the magnitude of the induced dipole produced at the nano-object. A comparison between the highest (27 nN) and lowest (3.4 nN) near-field forces along the dotted line profile revealed that the plasmonic enhancement factor ratio between the nanogap and the Ag surface in the dotted line was (27/3.4)^2^, suggesting a 63-fold higher enhancement at the nanogaps. It is interesting to note that such a high lateral resolution was obtained using a weak incident irradiation of <100 μW. Deformation of the near field arising from either unwanted chemical reactions or damage to the sample under high-irradiance incident light could distort the apparent plasmonic properties of the arrays.

**Fig. 3:**
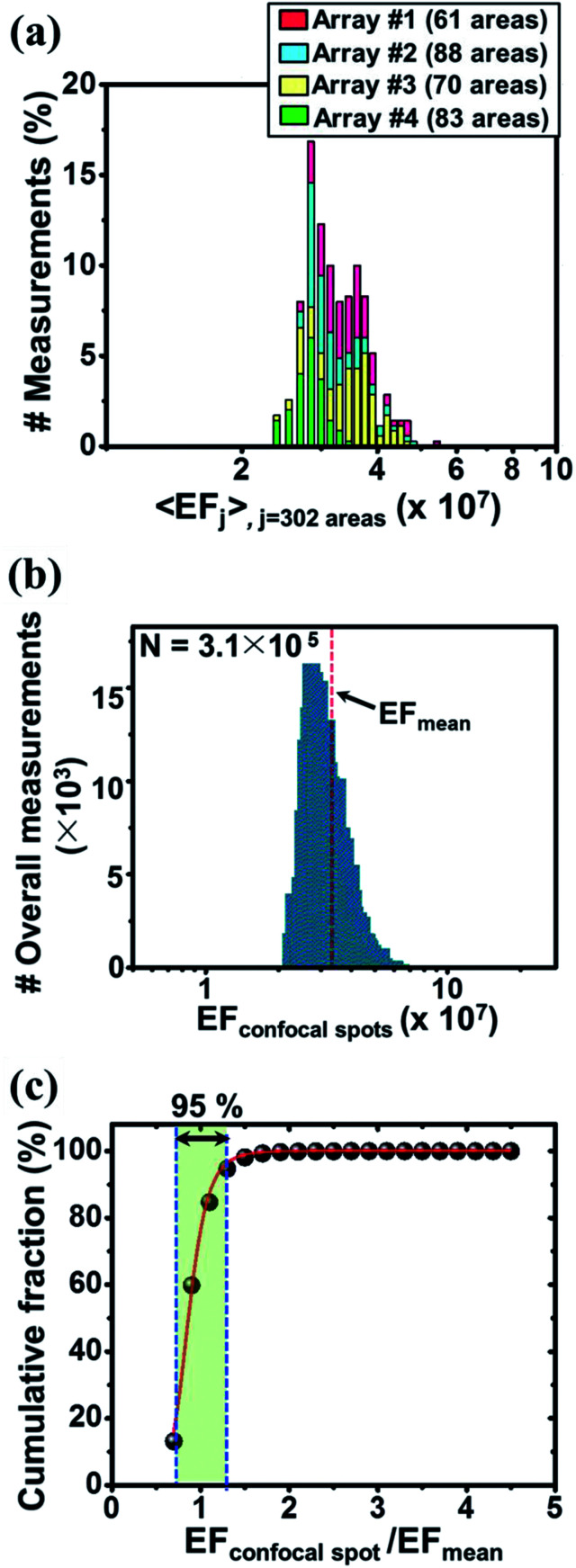
(a) The average SERS EF distribution histogram obtained from the 302 scanned areas over the four different SERS arrays fabricated using independent batch cycles. (b) The overall EF distribution obtained from all confocal points (3.1 × 10^5^ points; 32 pixels × 32 pixels × 302 areas). (c) Cumulative plot of the EF_confocal spot_/EF_mean_, obtained from the overall EF distribution shown in (b). A logistic function, *y* = *A*/(1 + (*x*/*x*_0_)^−*p*^), was employed to fit our data. The parameter *A* was set to 100 and represented the total fraction of the examined confocal spots (3.1 × 10^5^ spots). The EF_confocal spot_/EF_mean_ values were densely populated (95%) over the narrow range 0.67–1.3, as indicated by the large value of *p* (*p* = 8).

The central SERS EF values of the mapped areas were explored to investigate the signal uniformity both at the area-by-area and array-by-array levels (Fig. 3a). As described above, a scanned area was mapped using 32 × 32 confocal spots to give 1024 EF values. The central EF value for each mapped area was extracted using a Gaussian fit of the histogram formed from the 1024 EF values. An average 〈EF〉 histogram shown in Fig. 3a was then constructed using the central values of the 302 mapped areas. The standard deviation histogram of the central EF values was also obtained (ESI, Fig. S12a†). The mean EF value and the coefficient of variation (CV) obtained from the 302 central EF values were found to be (3.3 ± 0.51) × 10^7^ and 8.1%, respectively. The EF variations within each area were examined by exploring the ratio of the lowest and highest EF values within a mapped area (EF_max,*i*_/EF_min,*i*_; *i* = 302), and the mean magnitude ratio was found to be 2.4 (ESI, Fig. S12b†). The average 〈EF〉 histogram showed a narrow distribution at the area-by-area level. Four SERS arrays fabricated independently in four distinct batch cycles produced central EF values with a small CV of 9.8%. Such high process reproducibility was attributed to the simple two-step fabrication process. The uniformity of the EF distribution was thoroughly characterized by constructing an overall histogram (Fig. 3b) assembled from a total of 3.1 × 10^5^ confocal spots obtained from 32 pixels × 32 pixels × 302 mapped areas. The lowest and highest EF values were found to be, respectively, 2.2 × 10^7^ and 15 × 10^7^, indicating a markedly narrow EF distribution range of <10^1^.

To quantitatively access the fraction of hot spots present, a logistic function, *y* = *A*/(1 + (*x*/*x*_0_)^−*p*^) typically employed to describe population growth in economics and biology was applied (Fig. 3c). The parameter A was fixed to 100, corresponding to the overall fraction of the confocal spots examined, 3.1 × 10^5^ spots. The cumulative plot extracted from the overall EF histogram shown in Fig. 3b was fit to this function. This plot was advantageous in that it directly visualized the specific fractions of hot spots. The EF_confocal spot_/EF_mean_ values were densely populated (95%) over a narrow range (0.67–1.3), as indicated by the large *p* value (*p* = 8). A small *p* value, conversely, would have reflected a low density of hot spots, indicative of the poor performance of the sensing array with respect to uniform plasmonic response generation. This simple plot provides a useful tool for quantitatively comparing the signal uniformities of SERS arrays fabricated using different methods. In our case, all EF_confocal spot_/EF_mean_ values fell within a narrow range, 0.67–4.6. This persistent challenge of obtaining uniform SERS responses reflects the fact that nanoscale changes in the gap distances, locations, and orientations of the plasmonic hot spots, the surface energy, and the complicated interplay among these factors have resulted in uncontrollable SERS signal instabilities. A significant increase in the areal density of nanogaps over a large surface area is required to compensate for the “intrinsically random nature” of hot spot generation to enable reliable and sensitive SERS detection.

## Conclusions

In summary, we demonstrated that a wafer-scale nanolithography-free SERS substrate with dense nanogaps generated a sensitive and uniform SERS response. The plasmonic array features 120 particles per μm^2^ globular Ag nanoparticles. Such dense nanogaps led to a high SERS EF of 3.3 × 10^7^ and a narrow SERS EF distribution with CV of 8.1%. The local near-field forces were visualized using PiFM. These direct measurements yielded the spatial resolution of 3.1 nm. The magnitude of the near field at the nanogaps was found to be a 63-fold higher than that on the Ag surfaces, indicating the effectiveness of the nanogap in producing an enhanced plasmonic field. Ultimately, the quantitative measurement of the near-field distributions will provide practical guidelines for the rational design of metal nanostructured arrays with controlled and optimized optical signals for use in a broad range of nanoplasmonic-based imaging and sensing applications.^29–35^

## Experimental

### Fabrication of the plasmonic nanostructured array

The PET pretreatment was performed by exposing the PET surface to an Ar plasma in a custom-built radio frequency (RF) ion etching system employing a 13.56 MHz capacitively coupled plasma supply. The PET substrate was attached to a plate electrode positioned 15 cm above the showerhead and pretreated with Ar plasma at a plasma power of 200 W (1.1 W cm^−2^) for different periods of time. The inlet Ar flow rate and the reactor pressure were fixed at, respectively, 50 standard cubic centimeters per minute (sccm) and 23 Pa during the pretreatment process. The subsequent Ag deposition was prepared using a direct current (DC) magnetron sputtering system (Flexlab System 100, A-Tech System Co.) with a 4 inch Ag target (Williams Advanced Material Inc.) at a DC power of 50 W (0.13 W cm^−2^). Prior to sputtering, the sputtering chamber was evacuated to a base pressure of 1.3 × 10^−4^ Pa. The pressure was increased to 0.4 Pa by introducing Ar gas at a flow rate of 45 sccm. The Ag deposition rate was measured to be 0.24 nm s^−1^.

### Measurements and characterizations

Plane and cross-sectional images of the arrays of polymer protrusions and Ag nanoparticles were obtained using ultrahigh resolution (UHR) SEM (S-5500, Hitachi) techniques at the Korea Basic Science Institute (Jeonju, South Korea). Low accelerating voltages of 2–5 kV were applied using the electron beams to avoid thermal damage to the PET substrate. The absorption spectra of the SERS substrates were obtained by measuring the transmittance and reflection of the substrate using UV-visible spectrometry (Cary 5000, Varian). A microRaman microscope (Horiba Jobin Yvon, LabRAM HR) equipped with a 514.5 nm excitation source using 50× objective lens (numerical aperture (NA) = 0.5) was employed to measure the SERS signals of the adsorbed benzenethiol molecules. The incident power and accumulation time were 150 μW and 10 seconds, respectively. The SERS spectra were measured at 9 positions across the entire 4 inch surface area. A VistaScope AFM from Molecular Vista was employed for PiFM measurements. A 532 nm laser was coupled to measure the field distribution of the SERS. The laser beam was side-illuminated to the sample with an angle of 30° by a parabolic mirror whose NA is 0.4. The microscope is operated in non-contact/tapping mode. The Au coated AFM cantilevers (Nanosensors, PPP-NCH-Au) with 300 kHz fundamental resonance were used, and the radius of each AFM tip was about 25 nm. The fundamental resonance is typically around 300 kHz and the second resonance is around 1.8 MHz. The topography is probed by the second resonance of the cantilever and the PiFM is measured at the fundamental resonance. The PiFM is operated as the direct mode to quantitatively reconstruct the photo-induced force, which modulates the laser beam at the detection (fundamental) frequency of the cantilever, as *f*_m_ = *f*_1_ = 300 kHz, by using the acousto-optic-modulator.

### The average EF estimation

The average EF of the SERS array was calculated using following equation,SERS EF = (*I*_SERS_/*I*_Raman_) × (*N*_Raman_/*N*_SERS_)where *I*_SERS_ and *I*_Raman_ represent the scattering intensities, corrected according to the incident power and the acquisition time used to measure the SERS and normal Raman signals of the adsorbed benzenethiol molecules in the presence and in the absence of the Ag nanoparticles, respectively. Likewise, *N*_SERS_ and *N*_Raman_ indicate the number of molecules responsible for the *I*_SERS_ and *I*_Raman_, respectively. *N*_SERS_ and *N*_Raman_ were estimated to be 8.48 × 10^−22^ and 8.18 × 10^−16^ moles, respectively, and were present within a diffraction-limited spot volume having a 360 nm FWHM diameter and a confocal volume of 8.4 × 10^−14^ cm^3^, respectively. The confocal volume (*V*_c_) was estimated using the equation, *V*_c_ = π^3/2^*kw*^3^, where *k* (=2.33*n*/NA, *n* is the index of refraction of the medium) and *w* are the vertical and lateral resolutions of the incident light at the confocal spot, respectively. Here, all benzenethiol molecules adsorbed onto the Ag particles were assumed to contribute to the observed SERS signals. The resultant average SERS EF was 3.3 × 10^7^.

## Contributions

J. Y. and H. L. conceived the experiment. C. W. M. performed the fabrication of SERS array. J. J., W. A. M. and D. B. N. performed PiFM measurements. J. H. S. performed FDTD simulation. T.-S. B. performed FIB-SEM measurements. D.-K. L., H. M. K., N. H. K., S. H. N. and J. K. performed SERS measurements. M.-K. S., D. H. K., S.-G. P. and Y. D. S. supervised the work and discussed the results. J. Y., H. L., J. H. S. and S.-G. P. wrote the paper, with input from all authors.

## Conflicts of interest

There are no conflicts to declare.

## Supplementary Material

RA-008-C7RA13322G-s001
