# Supplementary material for: Fabrication and near-field visualization of a wafer-scale dense plasmonic nanostructured array
Source: RSC Adv. 2018 Feb 8;8(12):6444–51. doi: 10.1039/c7ra13322g (PMC9078216; doi:10.1039/c7ra13322g)
Supplement: RA-008-C7RA13322G-s001 [file RA-008-C7RA13322G-s001.pdf]

Electronic Supplementary Material (ESI) for RSC Advances.
This journal is © The Royal Society of Chemistry 2018

Supporting Information

Fabrication and Near-Field Visualization of Wafer-Scale Dense Plasmonic Nanostructured Array

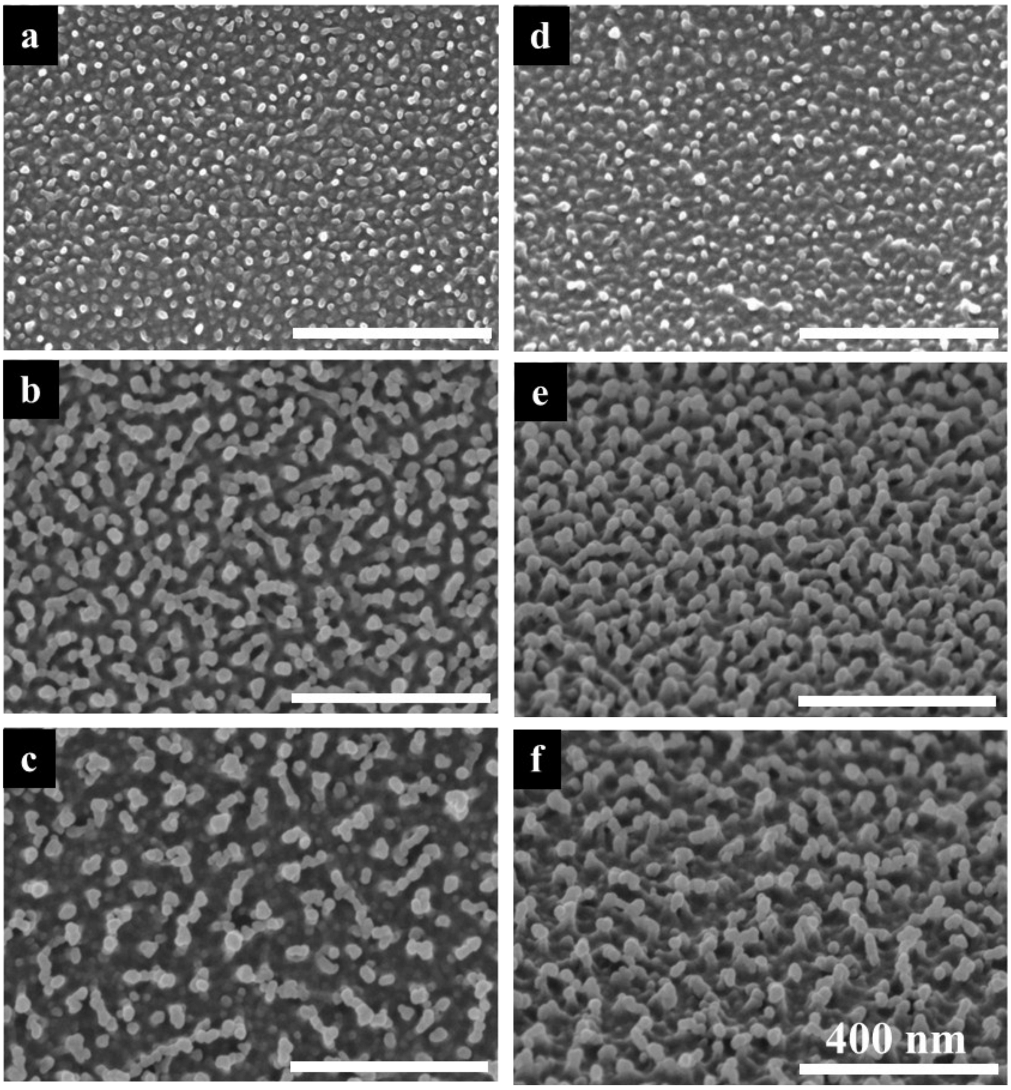

Fig. S1. (a–c) Plane and (d–f) tilted FE-SEM images of the polymer protrusions generated on the surface of the polymer (polyethylene terephthalate; PET) substrates treated under Ar plasma over various periods of time: (a, d) 20 seconds, (b, e) 60 seconds, and (c, f) 180 seconds.

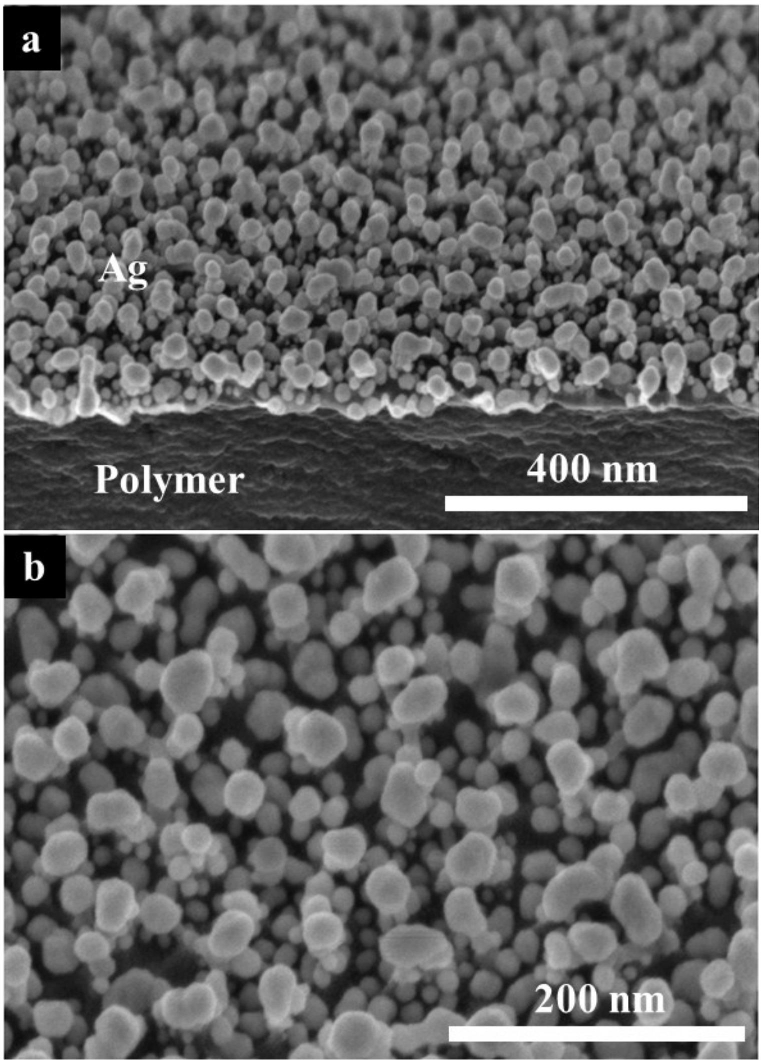

Fig. S2. (a) Tilted and (b) in-plane FE-SEM images demonstrating the growth competition among Ag nanoparticles on the polymer protrusions and on the recessed polymer surfaces between the protrusions. The treatment time for the polymer substrate and the Ag deposition thickness were chosen to be 60 seconds and 10 nm, respectively, to effectively show the preferential deposition of Ag onto the protrusions during the early stages of growth.

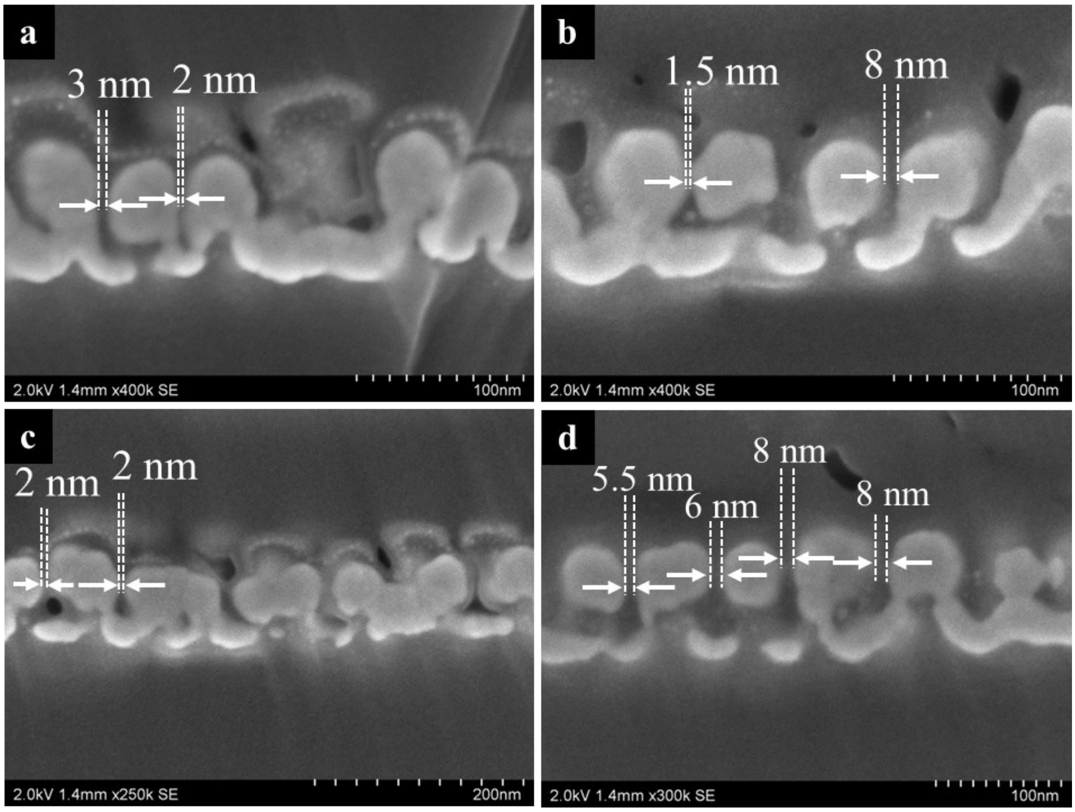

Fig. S3. Focused ion beam (FIB)-milled cross-sectional FE-SEM images of the nanogap-enhanced Raman scattering (SERS) array (a-d) displaying typical nanogaps formed between neighboring Ag particles. The magnified image of (a) is shown in **Fig. 1d**.

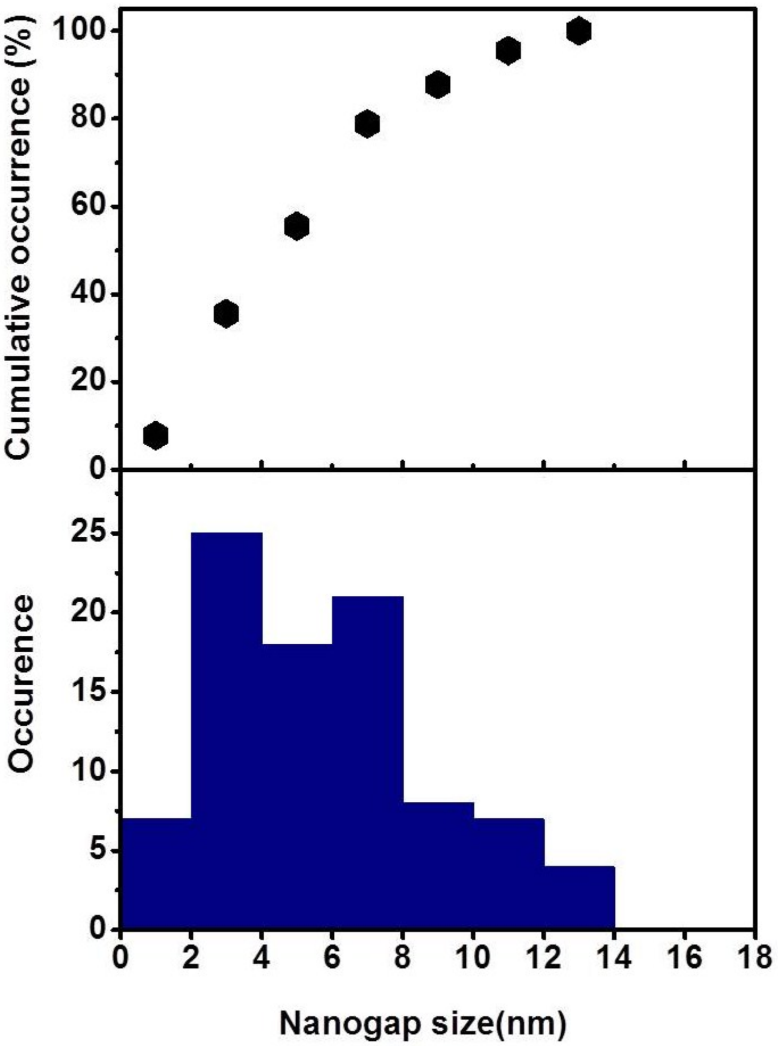

Fig. S4. The cumulative (upper panel) and normal (lower panel) representations of the nanogap size distribution, obtained from FIB-milled cross-sectional FE-SEM images. The mean gap distance of the investigated nanogaps was 5.6 ± 3.1 nm.

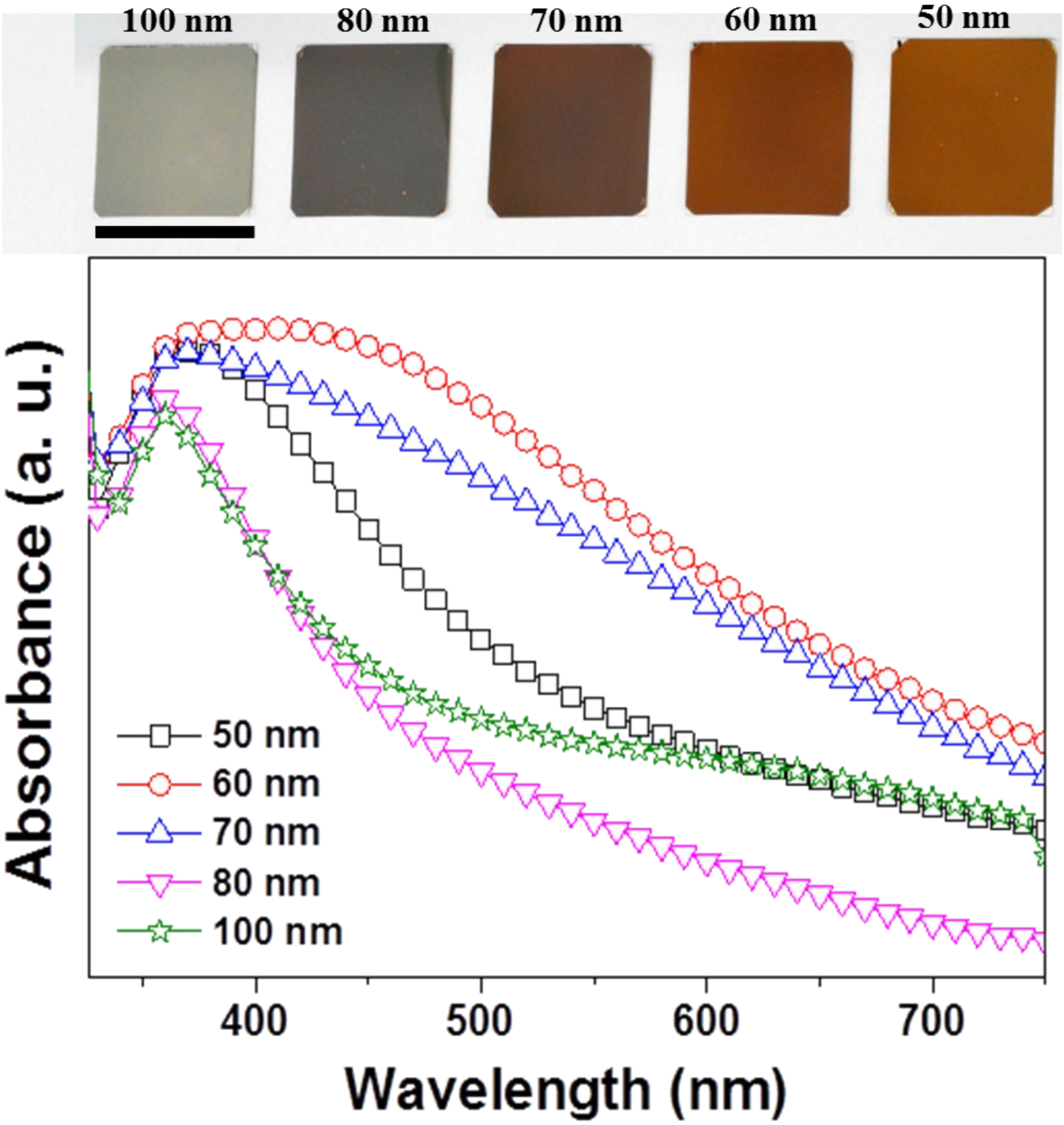

Fig. S5. UV-visible absorption spectra of substrates prepared with arrays of Ag nanoparticles with different diameters ranging from 50 to 100 nm. The LSPR peak in the 60 nm Ag particle array (red) was red-shifted and significantly broadened at longer wavelengths, indicating strong plasmonic couplings between adjacent particles. Scale bar is 2 cm.

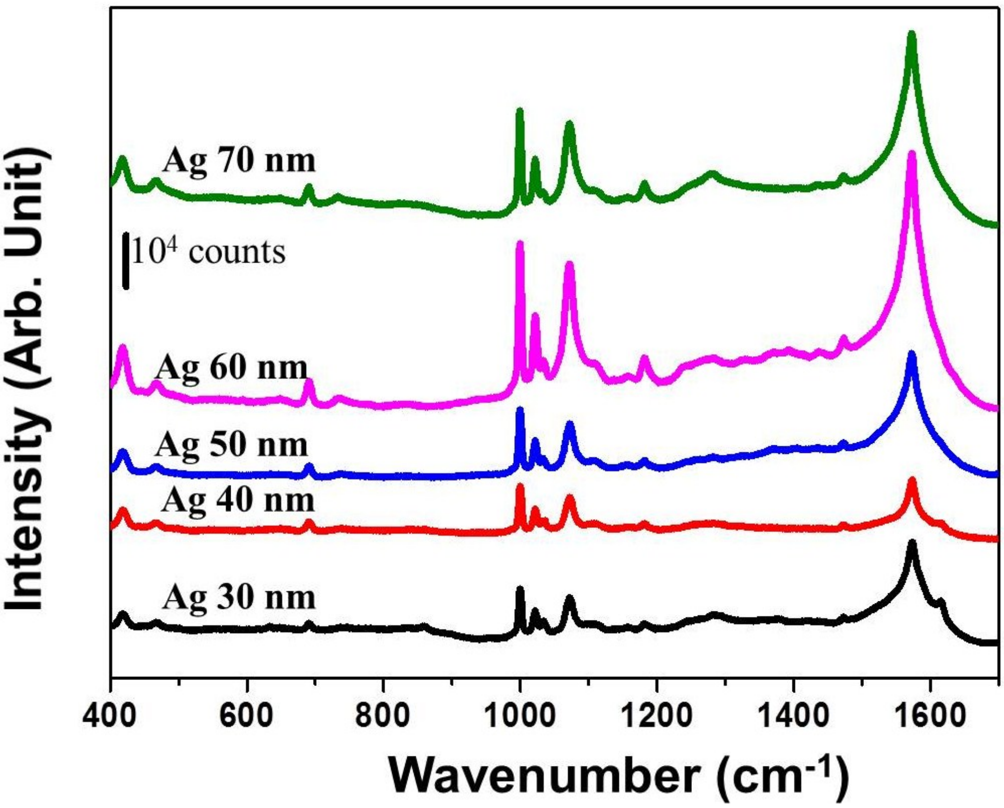

Fig. S6. The measured SERS spectra of the benzenethiol molecules (2 μM) adsorbed onto SERS arrays prepared with arrays of Ag nanoparticles having different diameters. A microRaman microscope with a 514.5 nm excitation was employed for the SERS measurements. The incident power and accumulation time were 1.5 mW and 30 seconds, respectively.

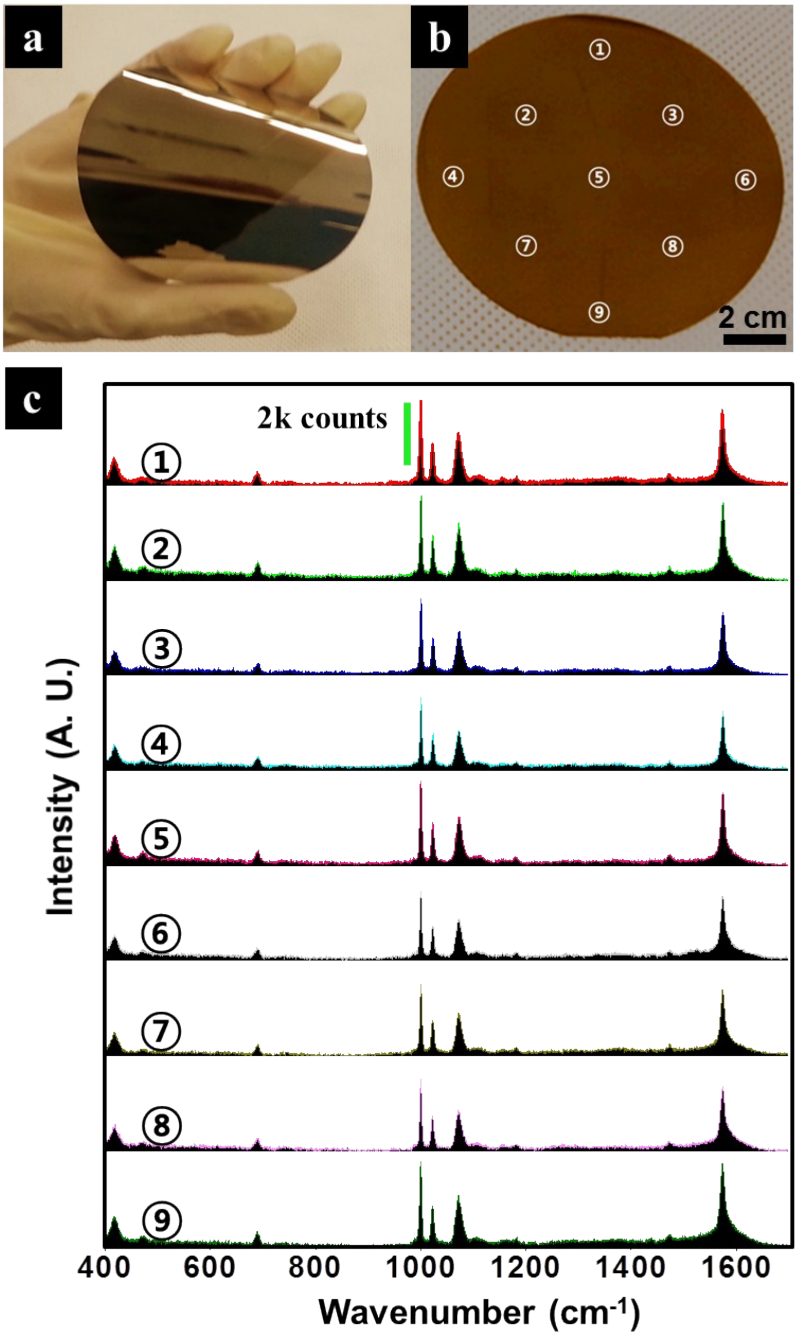

Fig. S7. (a-b) Photograph of a 4 inch flexible SERS array supporting 60 nm Ag nanoparticles. (c) SERS spectra of 2 μM benzenethiol molecules, measured at nine different positions shown in (b). A Raman microscope with a 514.5 nm excitation source was employed, and the incident beam size was 1.3 μm. The incident power and accumulation time were 150 μW and 10 seconds, respectively.

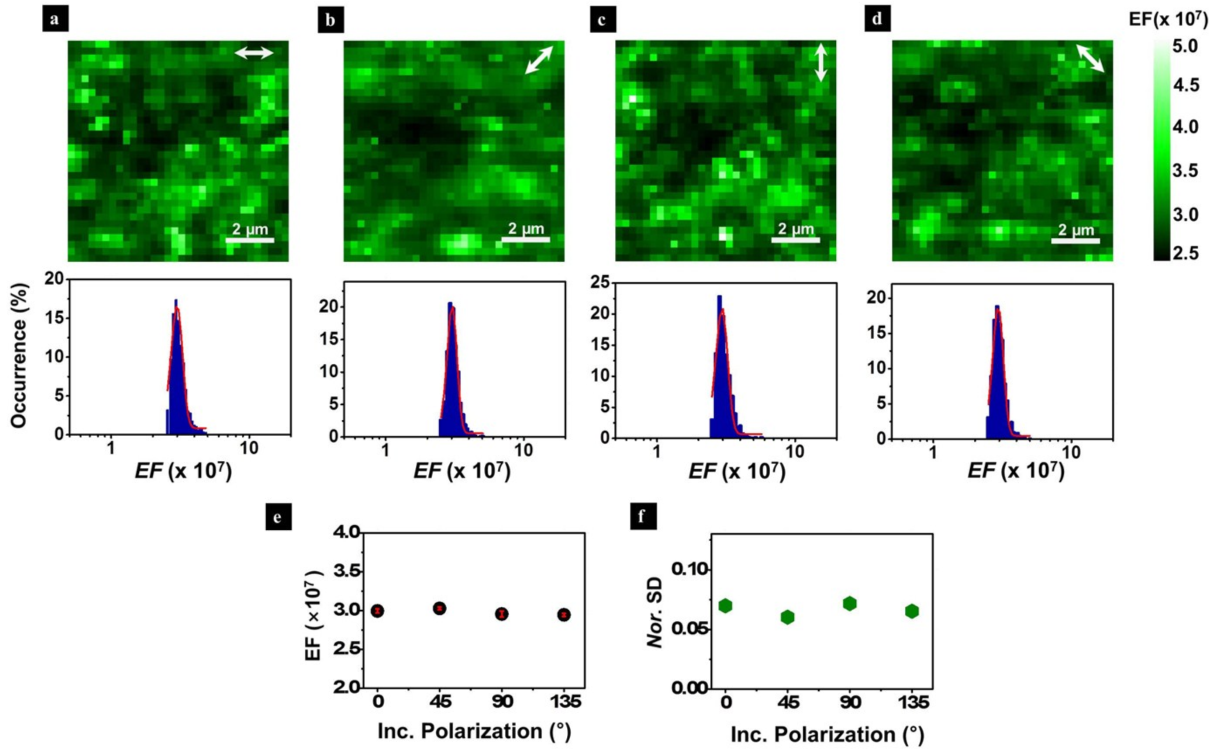

Fig. S8. (a)–(d) Spatially mapped EF images (top panel) and the corresponding EF distributions (middle panel) collected using incident polarization angles stepped in 45° increments. The EF values (e) and their standard deviations (f) were obtained by Gaussian fitting.

<PiFM technique>

PiFM, in principle, detects the axial near-field force between an optically-driven induced dipole at a nano-object (nanogaps and Ag particles, in our case) and its mirror image dipole created at the tip apex of a metal-coated atomic force microscopy (AFM) probe.[1–4] The near-field gradient force between two interacting induced dipoles is inversely proportional to the forth power of the distance between the induced dipole and the image dipole, r^{-4} (**Figure** S10). The z-component (F_z), parallel to the tip apex and proportional to the magnitudes of both the x and y components of the measured near-field gradient force could be expressed as

$$< F_z >= -\left(3 \cdot \alpha_{tip} \cdot \alpha_{nano-object}/2\pi r^4\right)\left|E_{inc,z}\right|^2 \qquad (1)$$

where $\boldsymbol{\alpha}_{tip}$ and $\boldsymbol{\alpha}_{nano\text{-}object}$ are the polarizability tensors of a tip and a nano-object, respectively, in the absence of an optically induced field. The negative sign indicates an attractive near-field interaction. Note that because the near-field gradient force was measured (Fig. S10a), the PiFM did not intrinsically suffer from strong far-field background radiation as a result of direct excitation of the sample within the diffraction-limited illumination area. This technique is, therefore, particularly useful for plasmonic arrays with densely populated nanostructures. A simplified schematic diagram of the PiFM technique is presented in Fig. S10b. The incident light (532 nm laser light in our case) was modulated at a frequency f_m and generated the diffraction-limited focal spot on the surface of a bare SERS array using a parabolic mirror. The value of f_m was selected to be equal to the first mechanical resonance (f_0) of the cantilever, enabling sensitive detection of the photo-induced force signal. A corresponding topographic image was simultaneously obtained at the second natural resonance frequency (f_1) of the cantilever.

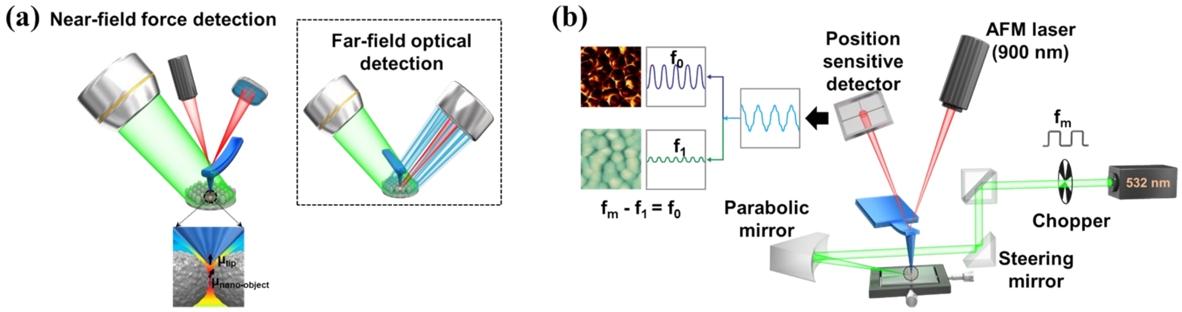

Fig. S9. Direct mapping of the dense plasmonic nanogaps using PiFM. (a) Simplified working principles underlying the near-field force-based PiFM and far-field radiation-based optical detection (insert). PiFM, by detecting the near-field force, whereas far-field optical detection essentially collected both the near-field signal (depicted as red), which was converted to radiation by the nanoprobe, and the far-field background (depicted as blue). (b) A schematic diagram of the PiFM instrumentation.

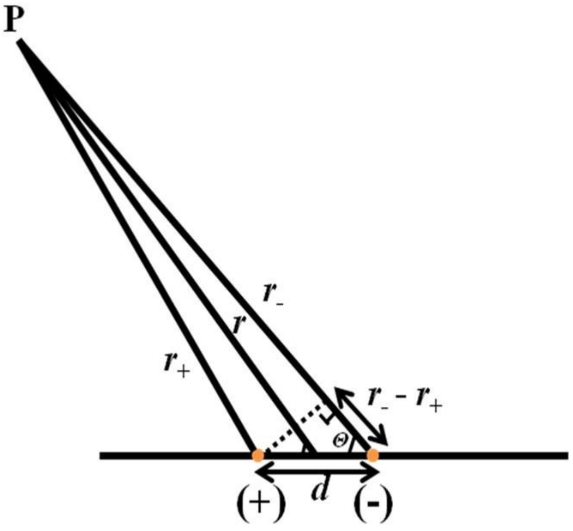

Fig. S10. **I**nduced (or electric) dipole moment with charge separation, *d*. The field point is sufficiently far from the dipole moment, so that the following assumption can be made: $r_+ \approx r\_ = r$

The electric potential generated by a dipole moment (μ) is

$$\varphi_D(r) = \frac{\sum_i q_i}{4\pi\varepsilon_0 \sum_i r_i}$$

$$= \frac{q_-}{4\pi\varepsilon_0 r_-} + \frac{q_+}{4\pi\varepsilon_0 r_+}$$

$$= \frac{q}{4\pi\varepsilon_0}\left(\frac{1}{r_+} - \frac{1}{r_-}\right) = \frac{q}{4\pi\varepsilon_0}\left(\frac{r_- - r_+}{r_+ \cdot r_-}\right)$$

$$= \frac{q}{4\pi\varepsilon_0} \cdot \frac{d\cos\theta}{r^2} = \frac{q \cdot d \cdot r\cos\theta}{4\pi\varepsilon_0 r^3} = \frac{(q \cdot d) \cdot r\cos\theta}{4\pi\varepsilon_0 r^3} = \frac{|\mu| \cdot |r| \cdot \cos\theta}{4\pi\varepsilon_0 r^3}$$

$$\therefore \varphi_D(r) = \frac{\mu \cdot r}{4\pi\varepsilon_0 r^3}$$

where ε_o is dielectric constant, $q\_$ and q_+ are the negative and positive charges of the dipole moment, and $r\_$ and r_+ are the distances from the negative and positive charges of the dipole moment to the field point at which observations are made, as shown above. We can then determine the electric field from the electric potential.

$$E_D(r) = -\frac{\partial \varphi_D(r)}{\partial r} = -\frac{\partial}{\partial r}\left(\frac{\mu \cdot r}{4\pi\varepsilon_0 r^3}\right)$$

$$= -\frac{\mu}{4\pi\varepsilon_0 r^3} + 3\frac{\mu \cdot r}{4\pi\varepsilon_0 r^4} r_{unit} \text{ using} \left(\frac{r}{|r|} = r_{unit}\right)$$

$$= -\frac{\mu}{4\pi\varepsilon_0 r^3} + \frac{1}{4\pi\varepsilon_0 r^3}\left(\frac{3\mu \cdot r \cdot r_{unit}}{r}\right)$$

$$= \frac{1}{4\pi\varepsilon_0 r^3}(3(\mu \cdot r_{unit}) \cdot r_{unit} - p)$$

$$\therefore E_D(r) = \frac{1}{4\pi\varepsilon_0 r^3}(3(\mu \cdot r_{unit}) \cdot r_{unit} - \mu)$$

The dipole–dipole interaction energy (U_{DD}) is given by

$$U_{DD} = -\mu_2 \cdot E_D(r) = -\mu_2 \cdot \frac{1}{4\pi\varepsilon_0 r^3}(3(\mu_1 \cdot r_{unit}) \cdot r_{unit} - \mu_1)$$

$$= \frac{1}{4\pi\varepsilon_0 r^3}[\mu_1 \cdot \mu_2 - 3(\mu_1 \cdot r_{unit}) \cdot (\mu_2 \cdot r_{unit})]$$

Finally, the electric force ($\boldsymbol{F}_2$) on μ_2, for example, may be expressed as follows.

$$F = -\nabla U_{DD} = \frac{3}{4\pi\varepsilon_0 r^4}\{\mu_1 \cdot \mu_2 - 5(\mu_1 \cdot r_{unit}) \cdot (\mu_2 \cdot r_{unit}) r_{unit}$$
$$+ (\mu_2 \cdot r_{unit})\mu_1 + (\mu_1 \cdot r_{unit})\mu_2\}$$

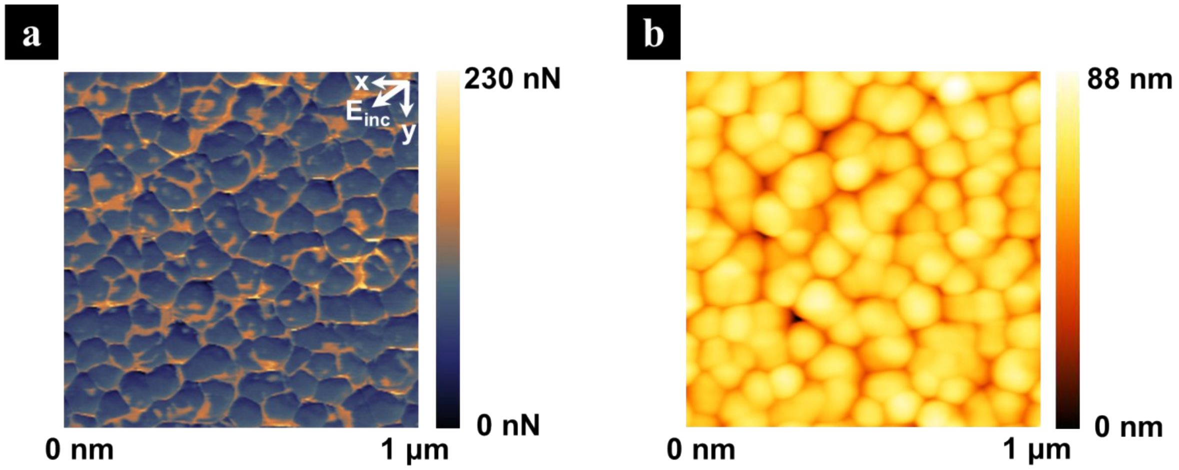

Fig. S11. (a) Photo-induced force image and the concurrently obtained (b) topographic image of a chosen area (1 × 1 μm²) in a bare SERS array.

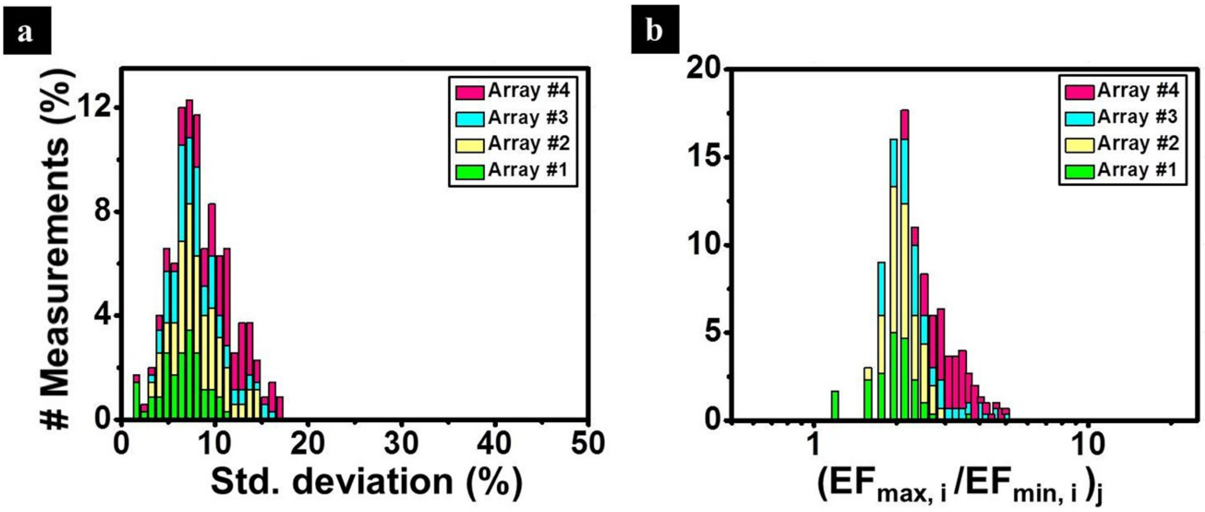

Fig. S12. (a) The standard deviation histogram of the central EF values measured from the 302 mapped areas. (b) The ratio distribution histogram of the highest and lowest EF values obtained from each of the 302 areas.

<References>

1. I. Rajapaksa, K. Uenal and H. K. Wickramasinghe, Appl. Phys. Lett., 2010, 97, 73121.

2. F. Huang, V. A. Tamma, Z. Mardy, J. Burdett and H. K. Wickramasinghe, Sci. Rep., 2015, 5, 10610.

3. J. Jahng, D. A. Fishman, S. Park, D. B. Nowak, W. A. Morrison, H. K. Wickramasinghe and E. O. Potma, Acc. Chem. Res., 2015, 48, 2671–2679.

4. J. Jahng, J. Brocious, D. A. Fishman, F. Huang, X. Li, V. A. Tamma, H. K. Wickramasinghe and E. O. Potma, Phys. Rev. B, 2014, 90, 155417
